# The impact of career expectation on employment anxiety of art students in higher vocational colleges during the COVID-19: A chain mediating role of social support and psychological capital

**DOI:** 10.3389/fpsyg.2023.1141472

**Published:** 2023-03-14

**Authors:** An Zhao

**Affiliations:** School of Art Design, Jiangsu College of Engineering and Technology, Nantong, China

**Keywords:** higher vocational art students, career expectations, employment anxiety, social support, psychological capital

## Abstract

**Objective:**

In the process of college students’ employment, psychological capital, and social support play a vital role.

**Methods:**

This study examined the relationship between career expectation and employment anxiety of Chinese vocational art college students (*N* = 634). Participants completed the Career Expectation Scale (CES), Employment Anxiety Scale (EAS), Psychological Capital Scale (PCS), and Social Support Scale (SSS).

**Results:**

(1) Vocational art students’ career expectation has a positive predictive effect on employment anxiety, social support, and psychological capital; Social support and psychological capital have negative predictive effects on employment anxiety. (2) Social support and psychological capital play a significant chain intermediary role between career expectation and employment anxiety, and there is a masking effect.

**Conclusion:**

These results are of guiding significance to the improvement of the employment quality of art students in higher vocational colleges and the employment consulting work in colleges.

## Introduction

The Chinese government proposed to speed up the construction of a modern vocational education system (Xue and Li, 2022), build a skilled society to promote employment, and let more young people realize their life value with their skills in 2021 (Li et al., 2021). With the slowdown of China’s economic growth, economic transformation and upgrading, the impact of COVID-19 on the economy and many other reasons (Han, 2022; Yu et al., 2022), the survival and development of various industries in China are facing great pressure, which also poses severe challenges to the employment of college graduates who are still expanding, especially the employment of vocational college graduates (Yang et al., 2022). At present, a lot of the public and some enterprises and institutions have low recognition of vocational education, they believe that the higher vocational students’ education is lower than that of undergraduate students, which means that the ability of higher vocational students is also lower than that of undergraduate students (Li, 2021). Meantime, the phenomenon of “emphasizing academic qualifications over technology” is common, which makes many vocational college graduates at a disadvantage or even suffer discrimination when seeking jobs (Zhang X., 2022). This kind of unfair treatment will make vocational students disappointed with their academic background, and repeated employment failures will also make them doubt their personal abilities, which, in turn, will produce anxiety and even dissatisfaction with society. As the cradle of the cultivation of practical technical talents in art, the employment situation faced by the graduates of higher vocational art majors is not optimistic. Due to the strong self-esteem of higher vocational art students, they have a high self-esteem, and they have higher expectations for the popularity of the recruitment company and the salary and treatment of the job. However, the actual situation is that the recruitment unit has high requirements for the work skills of higher vocational art students, but the salary is low; many recruiters are small or micro companies, with poor job stability and lack of promotion space. This gap between ideal and reality has caused nervousness, irritability, and other uneasiness in the employment process of higher vocational art students. This unease will grow as their graduation time approaches (Bolton, 2022; Li et al., 2022; Shi et al., 2022).

Employment anxiety refers to a kind of intense and persistent state anxiety caused by the uncertainty of employment results in the process of employment, which may cause a series of adverse physical and mental reactions (Unguren and Huseyinli, 2020; Chen and Zeng, 2021). The research shows that moderate employment anxiety can stimulate individual potential, promote self-ability, and actively participate in employment competition (Jang and Jin, 2020); However, high levels of employment anxiety can make individuals feel uneasy, fearful of helplessness, and even psychological barriers, which can affect physical and mental health and lead to employment failure (Joo, 2019). Previous studies on employment anxiety mostly started from specific methods such as alleviating employment pressure and psychological adjustment (Jeong, 2019; Lee and Ko, 2019), and rarely discussed the internal psychological mechanisms affecting employment anxiety. Research on employment anxiety is also mainly focused on undergraduate students, poor students, medical students, etc., and there is a lack of research on the group of art students in vocational colleges. In 2022, the number of students graduating from Chinese universities exceeded 10 million, a record high. At the same time, the downturn of China’s economy has made it difficult for many small and micro enterprises to survive, and even a wave of bankruptcy, and these small and micro enterprises are the main work of vocational college students. In addition, the normalization of the domestic epidemic and prevention and control measures have had a great impact on the social practice activities of students in vocational colleges. It can be seen that the record number of graduates and the pressure of China’s economic slowdown, coupled with the continuation of the epidemic, have aggravated the severe and complex employment situation of college students, bringing great anxiety and pressure to graduating college students, especially for graduates of vocational colleges with low educational levels. Based on this, this study relies on the relationship between vocational expectations, social support, psychological capital, and employment anxiety of students in vocational colleges, and whether the influence of social support and psychological capital on employment anxiety has a chain mediating effect. It is of great practical significance to prevent and alleviate the employment anxiety of art students in vocational colleges and universities under the state of large-scale public health emergencies and improve the mental health level.

### Literature review

Career expectation refers to the willingness and expectation of an individual to engage in a career in the future before entering the labor market. It emphasizes the internal motivation of individual career choice, and is also the external embodiment of individual occupational values (Hong et al., 2022). The study found that career expectation has a significant predictive effect on college students’ employment pressure, employment satisfaction, and employment quality (Bruthers and Matyas, 2020; Mcleod et al., 2021). At the same time, career expectation can affect employment anxiety by adjusting the positive coping style in college students’ employment activities (Seong and Francesca, 2020; Wu et al., 2020). College students who attach importance to internal value will feel the sense of urgency and anxiety to improve their ability, while college students who attach importance to external value will rely more on external forces (So and Ha, 2019; Choi and Jung, 2020). However, higher professional expectations will lead to more anxiety (Aydinli et al., 2019; Yang and Yang, 2022). This study therefore proposed the following hypothesis:

*H1*: The career expectation of art students in higher vocational colleges can positively predict employment anxiety.

As an extension of positive psychological ideas in the field of organizational behavior, psychological capital has received extensive attention from researchers in many disciplines. It is a positive psychological state or psychological energy displayed by individuals in the process of growth and development (Min and Minte, 2022; Xu and Wang, 2022). Psychological capital is closely related to the employment activities of college students, which can stimulate the internal employment potential of individuals, and change the cognition and attitude toward employment, thus increasing employment opportunities and enhancing employment success (Nimmi et al., 2021). Therefore, psychological capital plays a positive role in employment involving individual competence competition (Daniel et al., 2019; Lupsa and VÎrgĂ, 2020). In addition, the related research on psychological capital and employment anxiety shows that psychological capital has a positive impact on college students’ employment anxiety, that is, the higher the psychological capital, the lower the level of employment anxiety (Belle et al., 2022; Yang et al., 2022). Therefore, the second hypothesis was proposed:

*H2*: Psychological capital plays a mediating role in the process of the influence of career expectation on employment anxiety of vocational art students.

Social support refers to the resources provided by others to help individuals cope with the pressure they face (Lubis et al., 2022). Because social support comes from many aspects, including material support, spiritual inspiration, and help from relatives and friends. For art students in higher vocational colleges, the choice of occupation, where to obtain employment, how to obtain employment and how to realize their self-value after employment should be carefully considered, and more importantly, they need to rely on relevant social support resources to complete (Zhang Y.Z., 2022). It can be seen that social support plays a very important role in college students’ employment. Research on social support and employment anxiety shows that there is a significant negative correlation between social support and employment anxiety of college students (Kautish et al., 2021; Noman et al., 2021); In the face of employment pressure or employment choices, the more abundant social support resources for college students, the lower the level of employment anxiety (Yamashita and Kazama, 2018; Jang and Jin, 2019). In addition, the social support buffer theory points out that, on the one hand, social support can directly reduce individual pressure and improve social adaptability (Yang et al., 2020; Wang and Li, 2022), and on the other hand, it can improve anxiety and tension and promote physical and mental health through mediation effect (Unguren and Huseyinli, 2020; Chen and Zeng, 2021). Therefore, individuals with higher levels of social support have higher levels of mental health. Based on the above research results, the third and the fourth hypothesis were proposed:

*H3*: Social support plays a mediating role in the influence of career expectation on employment anxiety of vocational art students.

*H4*: Social support and psychological capital play a chain intermediary role in the impact of career expectations on employment anxiety of vocational art students.

## Materials and methods

**Figure 1:**
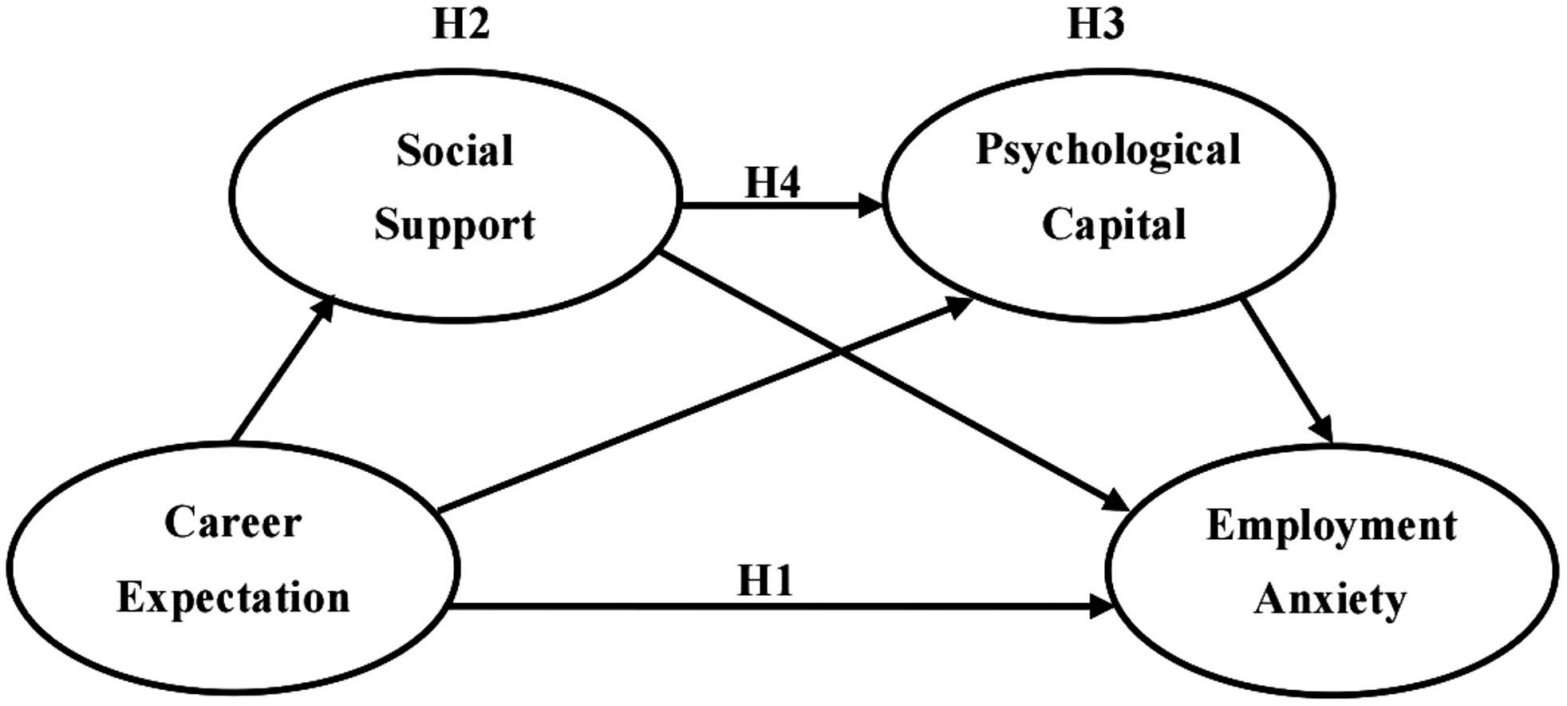
Hypothetical model.

According to the literature review and research purpose of this study, a hypothesis model was proposed (Figure 1). Social support and psychological capital were important influencing factors between career expectation and employment anxiety, and there was a chain relationship between social support and psychological capital.

### Participants

With the informed consent of the survey participants, the questionnaire was distributed online through the professional survey platform Questionnaire Star from March 10 to 20, 2022, and effective screening sampling was conducted. The survey object of this online questionnaire is the recent graduates of five vocational colleges in Jiangsu Province who have not signed formal employment contracts (age = 22–24, mean age = 23.35, SD = 1.316 years). A total of 634 questionnaires were distributed. After excluding the questionnaires with missing answers and repeated answers, 619 graduates were finally selected as research participants. The effective rate of the questionnaire was 97.63%. Due to the gender structure of art students, there were more female participants than male participants in this study, 388 female students (62.70%) and 231 male students (37.30%).

### Research tools

#### Career expectation

The Career Expectation Scale (CES; Wu et al., 2020) was used to measure the status of Career expectations in three dimensions. There are eight items in the prestige position and stability dimension, eight items in the internal value dimension, and five items in the external value dimension. This version of the scale has been proved to have good reliability and validity among college students in Chinese Mainland (Wang et al., 2022). It uses a five-point Likert-type response mode with 5 = strongest and 1 = not strong. The higher the total score and subscale score, the higher the level of professional expectation. The Cronbach’ alphas of three subscales ranged from 0.87 to 0.91. The results of confirmatory factor analysis were χ^2^/*df* = 2.69, RMSEA = 0.05, CFI = 0.93, NFI = 0.92, GFI = 0.90.

#### Employment anxiety

The Employment Anxiety Scale (EAS; Zhang X., 2022) was used to measure the individual employment anxiety in four dimensions. Among them, there are seven items in the dimension of employment competition pressure, seven items in the dimension of lack of employment support, six items in the dimension of lack of self-confidence, and six items in the dimension of worry about employment prospects. This version of the scale has been proved to have good reliability and validity among college students in Chinese Mainland (Wang et al., 2022). It uses a five-point Likert-type response model with 5 = strongest, 1 = not strong. The higher the total score and subscale score, the higher the level of employment anxiety. The Cronbach’ alphas of four subscales ranged from 0.82 and 0.89. The results of confirmatory factor analysis were χ^2^/*df* = 2.17, RMSEA = 0.04, CFI = 0.97, NFI = 0.96, GFI = 0.96.

#### Psychological capital

The Psychological Capital Scale (PCS; Luthans and Broad, 2020) was used to measure the individual’s psychological state in four dimensions (resilience, optimism, self-efficacy, and hope), with five items in each dimension. This version of the scale has been proved to have good reliability and validity among college students in Chinese Mainland (Liu et al., 2022). It uses a seven-point Liker-type response model with 7 = very strong, 1 = very not strong. The higher the total score and subscale score, the higher the level of psychological capital. The Cronbach’ alphas of four subscales ranged from 0.84 and 0.90. The results of confirmatory factor analysis were χ^2^/*df* = 2.36, RMSEA = 0.07, CFI = 0.92, NFI = 0.91, GFI = 0.91.

#### Social support

The Social Support Scale (SSS; Costa et al., 2022) was used to measure the individual’s social support level in three dimensions (family support, friend support, and other support), with four items in each dimension. This version of the scale has been proved to have good reliability and validity among college students in Chinese Mainland (Tian and Shi, 2022). It uses a seven-point Liker-type response model with 7 = very strong, 1 = very not strong. The higher the total score and subscale score, the higher the level of psychological capital. The Cronbach’ alphas of three subscales ranged from 0.85 and 0.92. The results of confirmatory factor analysis were χ^2^/*df* = 2.55, RMSEA = 0.03, CFI = 0.97, NFI = 0.96, GFI = 0.96.

### Data analysis

SPSS 26.0 and its plug-in program were used to input, process, and statistically analyze the relevant data of this study. Methods include descriptive statistics, Pearson’s correlation analysis, and PROCESS macroanalysis (Model 6) to assess the chain mediating role of social support and psychological capital. The data were normalized by Z-score in this study. Since the data collected are mainly derived from participants’ self-reports, which may lead to common methodological biases, in order to further improve the rigor of the study, Harman univariate tests were used to test for biases in common methods before data analysis. The mediation effect test in this study adopts the bootstrapping method, according to BootLLCI, BootULCI to determine whether the interval contains 0, if it does not contain 0, the mediation effect is significant, and vice versa. The model sample size is set to 5,000 and the confidence interval (CI) is set to 95%.

## Results

### Common method deviation test

In this study, Harman’s single-factor was used to test the common method bias since we assessed career expectations, employment anxiety, social support, and psychological capital using measuring scale. The analysis results showed that there were 14 common factors with eigenvalues greater than 1, among which the explained variance of the first factor was 24.56%, which was less than the critical standard of 40%, indicating that there was no problematic common method bias (Podsakoff and Organ, 1986).

### Descriptive statistics

Table 1 presents the mean, standard deviation, and correlation coefficients for the study variables. Career expectation correlated moderately with employment anxiety (*r* = 0.574, *p* < 0.01), and social support (*r* = 0.392, *p* < 0.01), and slightly with psychological capital (*r* = 0.293, *p* < 0.01). Employment anxiety negatively correlated with social support (*r* = − 0.331, *p* < 0.01), and psychological capital (*r* = − 0.413, *p* < 0.01). Social support correlated moderately with psychological capital (*r* = − 0.413, *p* < 0.01), so this result meets the requirements of intermediary effect analysis. Meanwhile, all correlation coefficients are below 0.700, indicating that there is no multicollinearity in the data.

**Table 1:** Descriptive statistics and correlations of study variable.

	1	2	3	4
1. Career Expectations	—			
2. Social support	0.392^**^	—		
3. Psychological capital	0.293^**^	0.471^**^	—	
4. Employment anxiety	0.574^**^	−0.331^**^	−0.413^**^	—
Mean	3.919	4.772	3.773	6.682
SD	0.701	0.863	0.778	1.816

*N* = 634.

** *p* < 0.01.

### Main effect

**Figure 2:**
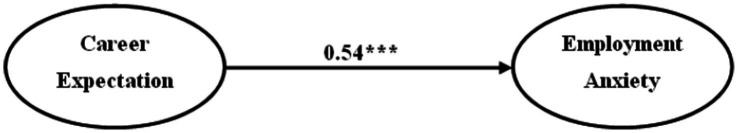
Main effect of career expectation to employment anxiety. ^***^p < 0.001.

When the main effect path was tested using SEM, the standardized regression coefficients of the main effect were between 0.27 and 0.54. The model demonstrated by the main effect path was suitable for the sample data: χ^2^ = 26.568, χ^2^/*df* = 2.214 (Schumacker and Lomax, 2004), RMSEA = 0.034 (Steiger, 1989; McDonald and Ho, 2002; Schumacker and Lomax, 2004), CFI = 0.982 and NFI = 0.973 (Bentler and Bonett, 1980; Hu and Bentler, 1999), GFI = 0.982, TLI = 0.972 (Doll et al., 1994; Schumacker and Lomax, 2004), and SRMR = 0.021 (<0.05; Jöreskog and Sörbom, 1989; Hu and Bentler, 1999). Career expectation explained 40.03% of the variance in employment anxiety levels (γ = 0.54, *p* < 0.001), thus supporting Hypothesis 1 (Figure 2).

### Structural model

**Figure 3:**
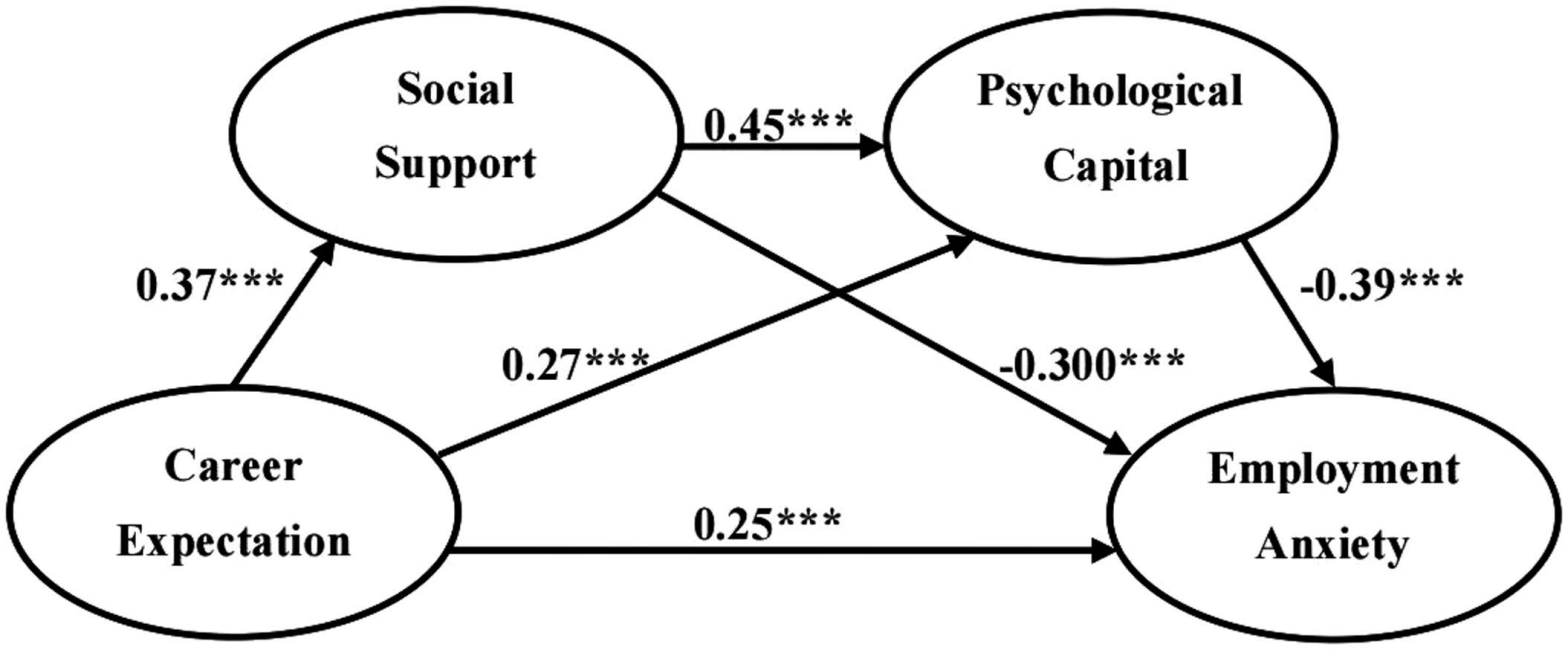
Structural model. ***p < 0.001.

The standardized regression coefficients of the main effect were between 0.25 and 0.45 in the SEM. The model demonstrated by the main effect path was a fit for the sample data: χ^2^ = 329.640, χ^2^/*df* = 2.658 (Schumacker and Lomax, 2004), RMSEA = 0.047 (<0.05; Steiger, 1989; Browne and Mels, 1990; McDonald and Ho, 2002; Schumacker and Lomax, 2004), CFI = 0.936 and NFI = 0.932 (Hu and Bentler, 1999), GFI = 0.925, TLI = 0.919 (Doll et al., 1994), and SRMR = 0.037 (< 0.05; Jöreskog and Sörbom, 1989; Hu and Bentler, 1999). Hypotheses 2–4, which involved mediating factors, constituted a structural model, as illustrated in Figure 3.

### Mediating effect

In order to test the hypothesis, bootstrapping was used to test the chain mediation model of the path between social support and psychological resources in occupational expectation and employment anxiety (Hayes, 2009). Table 2 shows the analysis results of bootstrap 95% confidence intervals (5,000 times), excluding 0 (Mackinnon et al., 2004); the direct impact of career expectation on employment anxiety was significant (γ = 0.253, *p* < 0.001). For the career expectation→social support→employment anxiety path, the bootstrap 95% confidence intervals (5,000 times) exclude 0 (γ = − 0.111, *p* < 0.001). For the career expectation→psychological capital→employment anxiety path, the bootstrap 95% confidence intervals (5,000 times) also exclude 0 (γ = − 0.105, *p* < 0.001). For the career expectation→social support→psychological capital→employment anxiety path, the bootstrap 95% confidence intervals (5,000 times) again exclude 0 (γ = − 0.007, *p* < 0.001), with the chain mediated effect discovered to be significant.

**Table 2:** Bootstrapping test result for chain mediating effects.

Total, direct, and indirect effect	Effect	Se	Boot95% CI	
			LLCI	ULCI
CE → SS → EA	−0.111	0.036	−0.216	−0.063
CE → PC → EA	−0.105	0.025	−0.221	−0.057
CE → SS → PC → EA	−0.007	0.011	−0.184	−0.039
Direct effects: CE → EA	0.253	0.049	0.301	0.395
Total indirect effect	−0.223	0.034	−0.278	−0.199
Total effect	0.476	0.068	0.397	0.522

CE, Career Expectation; SS, Social Support; PC, Psychological Capital; EA, Employment Anxiety.

## Discussion

### The impact of career expectations on employment anxiety

This study explores the influence of career expectations on employment anxiety of vocational art students and its internal mechanism. The results show that the career expectations of vocational art students have a significant positive predictive effect on employment anxiety, that is, the higher the level of career expectations of art students, the higher the degree of employment anxiety caused by this, which is completely consistent with previous research results and validates the first hypothesis of this study (Scholes and McDonald, 2021; Walters et al., 2022). In contrast, the human, material, and financial costs that art students need to spend in the process of growth and training are much higher than those of other majors, such as: art specialty training fees, learning equipment purchase fees (cameras, computers, painting tools, etc.), going out to collect style or visit exhibitions, etc. Influenced by the concept of “high investment will have high returns,” higher vocational art students have relatively high requirements for job salaries. At the same time, influenced by the idea that art is an elegant culture, art students often show a high self-esteem personality, and they have high expectations for the popularity or reputation of the employment unit, especially the salary and working environment. However, the actual level of positions, remuneration packages, and career development space that the job market (Watts et al., 2019; Mostafa et al., 2020) can provide is far from the initial psychological expectations of senior vocational art students (Maurud et al., 2022). The huge gap between ideal and reality should be one of the reasons for the employment anxiety of vocational art students.

### The independent mediating role of social support and psychological capital

This study explores that social support and psychological capital play a mediating role in the influence of career expectation on employment anxiety of art students in higher vocational colleges, which verifies the second and third hypotheses of this study, but both of these two mediating processes have a masking effect. First, career expectation can affect employment anxiety through psychological capital. The research results show that career expectation can significantly and positively predict psychological capital, which is completely consistent with the existing research results (Daswati et al., 2022; Obschonka et al., 2023). At present, many employers lack sufficient social recognition for art students in higher vocational colleges, which makes some excellent art students not be treated fairly and missed employment opportunities, affecting their future career development and life trend. At the same time, many art students in higher vocational colleges are not confident about their academic qualifications and professional abilities, and think they will lose in the employment competition with undergraduates (Hanife and Nazmiye, 2021). The internal and external dual pressure easily causes the graduates to have negative emotions such as tension and anxiety, which leads to a significant reduction in the internal confidence level of individuals, and even self-doubt. While career expectation has a positive impact on psychological capital (Collignon and Sajuria, 2021; Mei et al., 2022). When higher vocational art students show higher levels of psychological capital, they will have more confidence in their own professional abilities. They can maintain a positive attitude toward the unfair treatment of employers in the process of job hunting, constantly seek for deficiencies from their own aspects and strive to achieve employment, which greatly reduces the emergence of employment anxiety of vocational art students (Tatarko and Rodionov, 2017; Amy and Tony, 2020; Johnston and Cassidy, 2020). Therefore, this study confirms that psychological capital can alleviate employment anxiety. Secondly, career expectation can also affect employment anxiety through social support. The research results show that career expectation can significantly and positively predict social support, which is completely consistent with previous research results (Jia et al., 2018; Wu et al., 2019). Because most art students in higher vocational colleges lack clear goals and effective plans for their future career development, they will seek more social support resources to achieve successful employment if they have high expectations for their careers in the process of seeking employment. According to the theory of socialization, family is an important part of individual social support resources. Before art students in higher vocational colleges entered the employment market, their families were the earliest places for their socialization. The social class where their families lived, their parents’ professional background and interpersonal resources were important guarantees for their children’s successful employment (Baluku et al., 2020; Lowery and Cassidy, 2022; Zhao et al., 2022). Therefore, the ardent expectation of vocational art students will expand their social support system, improve the utilization of social support resources, and thus achieve successful employment. The impact of social support on employment anxiety is negative, which is completely consistent with previous research results (Amy and Tony, 2020; Yang et al., 2022), indicating that the higher the level of social support, the lower the degree of employment anxiety. When art students in vocational colleges encounter problems or difficulties in the process of employment, they are prone to mood swings that cause tension, anxiety, and worry. At this time, the support of society, family, friends, and other channels will help them improve their self-coping ability, face problems positively, alleviate anxiety, and effectively reduce employment anxiety.

### Chain mediating effect of social support and psychological capital

The study finds that social support and psychological capital play a chain intermediary role between career expectation and employment anxiety, which is basically consistent with previous research results (Kerksieck et al., 2019; Ren et al., 2019; Hye and Yeongmi, 2020), and also verifies the fourth hypothesis of this study. The main effect model of social support proposes that social support can not only alleviate the potential harm caused by stress events to individuals, but also have a positive impact on individual emotional experience and behavior patterns, so that individuals can enhance their sense of self-control, and even improve their physical and mental health by providing social support for individuals (Bruthers and Matyas, 2020; Martin et al., 2021). Therefore, the more abundant the social support resources are, the more positive the individual’s psychological state will be. It can be seen that social support can affect the individual’s emotional experience of stress and the way of coping with stress. High level of social support can promote the positive state of psychological capital.

## Conclusion

This study preliminarily reveals the relationship between career expectation and employment anxiety of art students in vocational colleges, and the role played by social support and psychological capital between them. Among them, career expectation has a positive predictive effect on employment anxiety, while social support and psychological capital have a masking effect here. This research result has important practical value for improving the employment quality of art students in higher vocational colleges. This requires higher vocational art students to make long-term plans for their careers in advance during their studies, not to focus on employment positions, but to use the employment platform to display their talents and continuously accumulate experience to improve themselves. Therefore, higher vocational art students should reasonably position their career expectations, overcome employment psychological barriers with the help of diversified social support, and actively face employment pressure by adjusting employment mentality, so as to reduce employment anxiety and promote employment quality. For example, vocational art students can find as many positions suitable for their career development as possible through the Internet, relatives and friends, and the talent market, and appropriately reduce the expectation of job salary. Higher vocational art studies also need to learn some employment interview skills, and show their professional expertise in the application process. At the same time, higher vocational art students should maintain a stable mood in the fierce competition for employment, by reducing the impact on themselves due to discrimination by recruiters. Of course, flexible employment is also another way out for higher vocational art students to find employment. In addition, university administrators, as student service providers, should provide scientific and effective employment guidance for students in the employment process. First of all, vocational colleges and universities should seek recruitment information from various relevant enterprises through multiple channels and provide it to students in a timely manner. Second, it is necessary to strengthen the professional concept of higher vocational art students and form the concept of “employment first, then career choice.” Third, for students with employment difficulties, schools should also provide assistance based on the student’s personal situation. In addition, due to the strong support of Chinese governments at all levels for college students in innovation and entrepreneurship policies and economy, vocational colleges and universities can also cultivate and support some potential innovation and entrepreneurship projects of higher vocational art students.

There are still several limitations in this study: (1) In terms of sample selection, the research subjects of this study are art graduates from five vocational colleges in Jiangsu. Due to the difference in the level of socio-economic development with the rest of the country and the uniqueness of the art profession, the scope of promotion of research results is limited, and the source of future research samples and professional types can be further expanded. (2) Due to the many factors affecting the employment anxiety of higher vocational art students, this study only explores the relationship between vocational expectations, social support, psychological capital, and employment anxiety of higher vocational art students, and can be further explored from the direction of personal ability and family socioeconomic status in the future.

## Data availability statement

The raw data supporting the conclusions of this article will be made available by the authors, without undue reservation.

## Ethics statement

Ethical review and approval was not required for the study on human participants in accordance with the local legislation and institutional requirements. The patients/participants provided their written informed consent to participate in this study.

## Author contributions

AZ: study concept and design, statistical analysis, and manuscript revise.

## Conflict of interest

The author declares that the research was conducted in the absence of any commercial or financial relationships that could be construed as a potential conflict of interest.

## Publisher’s note

All claims expressed in this article are solely those of the authors and do not necessarily represent those of their affiliated organizations, or those of the publisher, the editors and the reviewers. Any product that may be evaluated in this article, or claim that may be made by its manufacturer, is not guaranteed or endorsed by the publisher.
